# Exploring the therapeutic opportunities of potassium channels for the treatment of rheumatoid arthritis

**DOI:** 10.3389/fphar.2024.1286069

**Published:** 2024-05-09

**Authors:** Nikhil Eknath More, Rahul Mandlik, Sandip Zine, Vaibhavkumar S. Gawali, Angel Pavalu Godad

**Affiliations:** ^1^ SVKM’s Dr. Bhanuben Nanavati College of Pharmacy, Mumbai, India; ^4^ Department of Pharmaceutical Sciences and Technology, Institute of Chemical Technology, Mumbai, India; ^2^ Medical Affairs, Shalina Healthcare DMCC, Dubai, United Arab Emirates; ^3^ Charles River Laboratories, Cleveland, OH, United States

**Keywords:** Rheumatoid arthritis, ion channels, T lymphocytes, Kv1.3 channels, KCa3.1 channels, KCa1.1 channels, synoviocytes

## Abstract

Rheumatoid arthritis (RA) is a chronic inflammatory autoimmune disease that affects the synovial joint, which leads to inflammation, loss of function, joint destruction, and disability. The disease biology of RA involves complex interactions between genetic and environmental factors and is strongly associated with various immune cells, and each of the cell types contributes differently to disease pathogenesis. Several immunomodulatory molecules, such as cytokines, are secreted from the immune cells and intervene in the pathogenesis of RA. In immune cells, membrane proteins such as ion channels and transporters mediate the transport of charged ions to regulate intracellular signaling pathways. Ion channels control the membrane potential and effector functions such as cytotoxic activity. Moreover, clinical studies investigating patients with mutations and alterations in ion channels and transporters revealed their importance in effective immune responses. Recent studies have shown that voltage-gated potassium channels and calcium-activated potassium channels and their subtypes are involved in the regulation of immune cells and RA. Due to the role of these channels in the pathogenesis of RA and from multiple pieces of clinical evidence, they can be considered therapeutic targets for the treatment of RA. Here, we describe the role of voltage-gated and calcium-activated potassium channels and their subtypes in RA and their pharmacological application as drug targets.

## 1 Introduction

**FIGURE 1:**
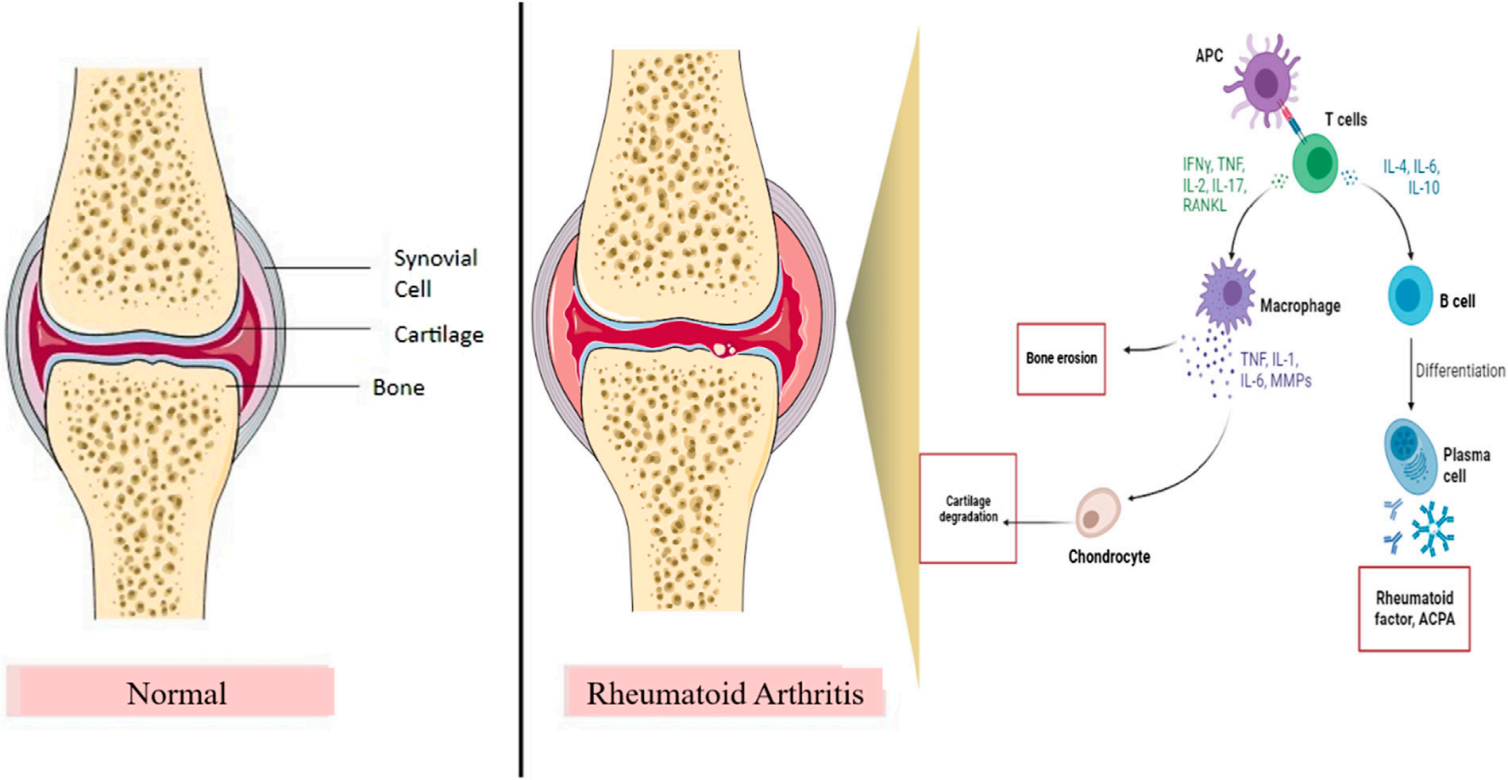
Role of the inflammatory pathway in RA pathogenesis. IL, interleukins; RANKL, receptor activator of nuclear kappa beta ligand; TNF, tumor necrosis factor; INF-*ϒ*, interferon-gamma; MMPs, matrix metalloproteinases; APC, antigen presenting cell. T cells and B cells are the important immune cells in the inflammatory pathway of RA. After binding of APC, B cells and T cells produce inflammatory cytokines and activate macrophages and plasma cells, thereby resulting in bone erosion and cartilage damage, which contribute to joint destruction and RA.

Rheumatoid arthritis (RA) is an inflammatory autoimmune disease (Andrei-Flavius and Simona Gabriela, 2021). The prevalence rate of RA was found to be 0.5%–1% worldwide (Arima et al., 2022). Primarily, it affects the linings of joints and is characterized by swelling, inflammation of the joints, redness, pain, arthralgia, synovitis, synovial hypertrophy, pannus formation, cartilage and bone destruction, and autoantibody production (Littlejohn and Monrad, 2018). The pathogenesis of RA involves the implication of the immune system and inflammatory pathways, which involve immune cells and inflammatory cytokines (Figure 1) (Dong et al., 2018). It is important for healthcare professionals to differentiate between clinical RA and osteoarthritis as both treatment and outcomes differ greatly between these two diseases (Littlejohn and Monrad, 2018). A differential diagnosis is required to identify the individual types of arthritis (Littlejohn and Monrad, 2018). The clinical diagnosis of RA is generally based on biomarkers such as the presence of the rheumatoid factor (RF), antibodies against citrullinated proteins (ACPA), erythrocyte sedimentation rate (ESR), and C-reactive protein (CRP) and multi-biomarker activity (MBDA) tests (Atzeni et al., 2017). Various disease-modifying anti-rheumatic drugs (DMARDs) are being used to treat RA, but they cause significant side effects such as weight loss, hypersensitivity, neuropathy, and hair loss (Aletaha et al., 2003). In addition, some patients do not respond to or tolerate the treatment (Watanabe et al., 2022). Hence, it is important to explore more targets and treatment therapies that may help in treating RA and are beneficial for the patients and healthcare society.

Ion channels came into the limelight for the treatment of autoimmune diseases as ion channels have the ability to establish membrane potential, ion concentration, and regulate immune response in immune cells (Chandy et al., 1985; Vinnenberg et al., 2021). Genetic alterations in ion channels contribute to the pathogenesis of several diseases. As such, there is no direct link between the mutations of potassium channels with RA pathogenesis. The primary potassium channels in human T cells, such as Kv1.3 and KCa3.1, play a crucial role in maintaining the ionic levels necessary for the healthy operation of T cells and the immunological response (Chirra et al., 2022). Studies identified that potassium channels regulate the immune cell function and control the RA-fibroblast-like synoviocyte (FLS) function, which is important in the pathogenesis of RA (Hu et al., 2012). In this review, we focus on the importance of voltage-gated and calcium-activated potassium channels in RA and how they can be utilized as therapeutic targets for the treatment of RA.

## 2 Immune system in RA

Both adaptive and innate immunity play an important role in the pathogenesis and development of autoimmune diseases such as RA (Trouw et al., 2013; Dong et al., 2018). The exact and complete role of innate immunity in the disease development of RA is not clearly known yet, but in the past two decades, emerging studies have shown that innate immunity also plays a critical role in RA (Dong et al., 2018). On the other hand, the impact of adaptive immunity on autoimmune diseases due to the ability to react to self-antigens is very well known, and evidence is present supporting this hypothesis, such as the presence of autoantibodies such as RF and ACPA (Yamada, 2022).

### 2.1 Adaptive immunity in RA

In RA, T cells and B cells are critically significant in autoimmunity and adaptive immunity. Studies have shown that in most cases of RA, there is a formation of ectopic lymphoid neogenesis (ELN), which comprises small portions of aggregates of T and B cells present in the synovium (Yamada, 2022). Although ELN is involved in RA progression, some researchers revealed that there is no correlation between ELN formation and local production of ACPA and RF (Cantaert et al., 2008). In addition, ELN is formed in various forms of arthritis, which excludes ELN from the race of diagnostic markers for RA (Yamada, 2022). Studies performed by researchers show conflicting data on the relationship between the presence of ELN and the treatment of RA (Yamada, 2022).

#### 2.1.1 B cells in RA

The increased number of autoreactive B cells plays an important role in the activation of autoreactive T cells (Wu et al., 2021a). KCa3.1 channels are expressed in B cells and regulate the migration and proliferation of B cells (Lin et al., 2022). B cells, as APCs, present their own antigens to CD4^+^ T helper cells, which results in an increase in the number of follicular helper T cells (Tfh) and peripheral helper T cells (Tph) in the synovium of RA patients (Lucas et al., 2020). Tfh and Tph then secrete CXCL13 and IL-21, which are important for the differentiation of B cells and the production of antibodies (Lucas et al., 2020).

B cells have a notable role in the cytokine secretion in RA (de Gruijter et al., 2022). B cells secrete cytokines such as TNF-α, interferon-γ(IFN-γ), IL-6, IL-1b, IL-17, and IL-10, which are involved in bone destruction and are related to the occurrence of the disease (Yanaba et al., 2008; de Gruijter et al., 2022). The amount of TNF-α produced by B cells in RA patients increases after the activation of Toll-like receptor 9 (TLR9) and CD40, which ultimately increases the expression of the receptor activator of the nuclear kappa beta ligand (RANKL) in the presence of IL-1β and promotes the formation of osteoclasts (Yeo et al., 2015). Similarly, TNF-α and CCL3 restrict bone formation in RA patients by inhibiting the differentiation of osteoblasts (Yeo et al., 2015).

#### 2.1.2 T cells in RA

T cells have an extensive role in the adaptive immunity and pathological management of RA (Yamada, 2022). T cells have a significant role in disease perpetuation as they are a source of pro-inflammatory cytokines and interact with synovial stromal cells (Scherer and Burmester, 2011; Komatsu et al., 2014). In T cells, the KCa3.1 channels initiate the expression of genes that promote T-cell activation and proliferation (Lin et al., 2022). Lines of evidence suggested that CD4^+^ T cells and CD8^+^ T cells play an important role in the progress and pathogenesis of RA (Jang et al., 2022). KCa3.1 channels support the migration of CD8^+^ T cells to the site of inflammation (Lin et al., 2022). In most of the cases, infiltration of a large number of CD4^+^ T cells is observed in the joint synovium and tenosynovium of RA patients, and these CD4^+^ T cells act as activation markers of T cells (Kaibara et al., 2008). CD4^+^ T cells help B cells in the production of autoantibodies and induction of inflammation at the joints (Yap et al., 2018). Due to the infiltration of CD4^+^ T cells and macrophages, it is considered a typical histological feature of RA synovitis (Li et al., 2021). T cells release IL-17, which is involved in the mobilization of neutrophils, activation of synovial fibroblast, and induction of RANKL expression.

In RA, it has been observed that there is a loss or functional deficiency of T-regulatory (T_reg_) cells (Jiang et al., 2021). The inhibition or decrease in the number of T_reg_ cells is associated with an increased immune response towards infectious pathogens (Jiang et al., 2021). The reports of a meta-analysis showed that the number of T_reg_ cells was decreased in peripheral blood (PB), while increased numbers were observed in synovial fluid (SF) (Morita et al., 2016). Synovial oxygen deficiency is one of the characteristics of RA; during this hypoxic environment, the synovial fibroblast induces T-cell differentiation, which leads to a decrease in the number of T_reg_ cells and an increase in the number of Th17 cells (Jiang et al., 2021).

### 2.2 Innate immunity in RA

Earlier, research was generally carried out to study the impact of adaptive immunity on the pathogenesis of RA, but nowadays, research has drifted towards studying the involvement of innate immunity in the pathogenesis of RA (Gierut et al., 2010). Various immune cells, such as monocytes, macrophages, dendritic cells (DCs), neutrophils, natural killer cells (NK), and innate lymphoid cells, contribute to RA pathogenesis via innate immunity (Bartlett et al., 2018; Chen et al., 2022). In the early stages of RA, the activation of innate immunity in the synovium serves as a key pathogenic mechanism that leads to the inflammation of joints (Gierut et al., 2010).

Macrophages and monocytes are important cells in innate immunity as they are the source of various cytokines responsible for maintaining the diseased condition, and the number of macrophages in the synovial tissue is considered the most reliable marker of disease severity in RA (Tak and Bresnihan, 2000). In tissues of RA patients, M1-like macrophages overexpress major histocompatibility complex (MHC) class II molecules, which are associated with increased inflammation and tissue damage (Edilova et al., 2021; Weyand and Goronzy, 2021).

DCs are another type of immune cell that are involved in RA and are divided into two subsets: classical DCs (cDCs) and plasmacytoid DCs (pDCs) (Edilova et al., 2021). By secreting a large number of cytokines such as TNF, IL-6, IL-1, and IL-12 and differentiation factors such as the macrophage colony-stimulating factor (M-CSF) and fibroblast growth factor (FGF), both cDCs and pDCs contribute to RA pathogenesis (Saferding and Blüml, 2020). In RA, neutrophils are the most abundant leukocytes in the inflamed joints and are the first to reach the synovium (Cecchi et al., 2018). Fcγ receptors are present on the membrane of neutrophils, and with the help of these receptors, neutrophils bind to the immune complexes, resulting in their degranulation and production of reactive oxygen species (ROS) (Cecchi et al., 2018). This enhanced ROS generation leads to endothelial dysfunction, tissue injury, DNA damage, oxidation of lipids and proteins, and immunoglobin mutations (Edilova et al., 2021).

## 3 Translational studies supporting the role of ion channels in rheumatoid arthritis

There are numerous human cell studies available in the literature that suggest the potential role of ion channels in the pathogenesis of RA. Mutation and functional changes in the ion channels lead to malfunctioning of the immune system, and this malfunctioning promotes RA. Hu *et al.* collected the FLSs of 12 RA patients to study the expression of the KCa1.1 channel. They found that the KCa1.1 channel is expressed on the plasma membrane of the FLSs and accounts for K^+^ current conduction in FLSs (Hu et al., 2012). The KCa1.1 channel is also involved in regulating the β_1_-integrin function (Tanner et al., 2017a). Similar results were obtained from Pethő et al.’s study, where they showed that β subunits of KCa1.1 are expressed on the plasma membrane of FLSs (Pethő et al., 2016). Tanner *et al.* studied the interaction between KCa1.1 and Kv1.3 channels and FLSs. It was found that the KCa1.1 channel regulates the FLS-mediated activation and proliferation of effector memory T (T_EM_) cells. KCa1.1 also regulates the surface expression of MHC II (Tanner et al., 2019). Beeton *et al.* studied the expression of the Kv1.3 channels with autoreactive T cells in RA patients. They found that there is an upregulation of Kv1.3 channels with T_EM_ cells. The expression of the Kv1.3 channel was found to be lower in the T_EM_ cells in healthy individuals. They tried to block the Kv1.3 channels with the help of selective Kv1.3 blockers, such as PAP-1 and SL5. It was found that the blockade reduces the activation and proliferation of T_EM_ cells. Similar results were obtained in the study performed by Tanner et al., where they demonstrated the role of KCa1.1 and Kv1.3 in regulating T_EM_ cells in RA (Beeton et al., 2006).

## 4 Potassium channels’ impact on immune function and RA

Ion channels act by maintaining the membrane potential through the influx or efflux of particular ions responsible for hyperpolarization or depolarization (Ehling et al., 2011). Because RA is an autoimmune disease, ion channels, specifically voltage-gated and calcium-activated potassium channels, are very important in maintaining the immune response and pathogenesis of RA.

**FIGURE 2:**
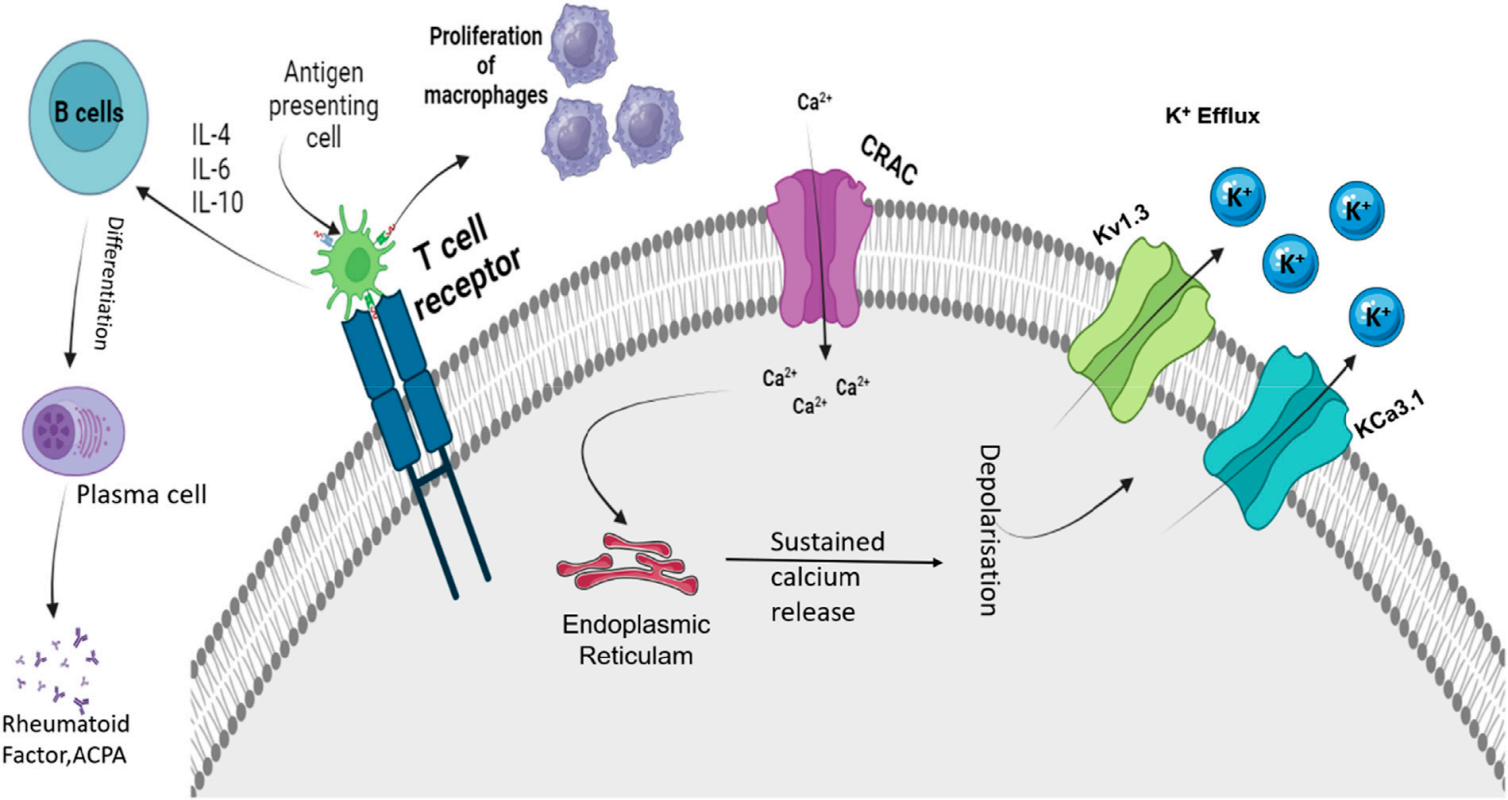
Roles of Kv1.3 and KCa3.1 in regulating depolarization shift that is important for immune function. APC, antigen presenting cell; IL, interleukin; ACPA, antibodies against citrullinated proteins; Kv1.3, voltage-gated potassium channel 1.3; KCa3.1, calcium-activated potassium channel 3.1; and CRAC, calcium-release-activated Ca^2+^ channel. Activation of TCR due to APCs activates the CRAC channel that causes an influx of Ca^2+^ ions. These Ca^2+^ ions activate ER stores and cause sustained release of Ca^2+^. This causes the activation of calcium-mediated and voltage-sensitive potassium channels such as Kv1.3 and KCa3.1. Upon activation, these channels cause the efflux of K^+^ ions. The influx of Ca^2+^ regulates the proliferation, activation, and differentiation of various immune cells.

As discussed in Section 2.1.2, T_reg_ cells play a significant role in RA pathogenesis, and proper ion regulation is required for the regulation and effector function of a cell (Varga et al., 2009; Kotschenreuther et al., 2022). T_regs_ are critical contributors to immune tolerance, and defects in their function are associated with autoimmune diseases such as RA (Vinnenberg et al., 2021). The potassium channels, such as voltage-gated K^+^ channel Kv1.3 and calcium-activated K^+^ channel KCa3.1, predominantly control membrane potential in T_reg_ cells (Vinnenberg et al., 2021). Both these channels are involved in the antigenic activation and proliferation of T cells, and each channel expresses differently in each T-cell subtype (Vinnenberg et al., 2021). Studies indicated the high Kv1.3 channel activity in the pathogenesis of RA, and dominant KCa3.1 channel expression was observed in Th1 cells, which is closely associated with autoimmunity (Vinnenberg et al., 2021). Similar to T_regs_, DCs are involved in the progression of RA as they are specialized APCs and are engaged in both specific immunity and immune tolerance (Veldhoen et al., 2006; Wehr et al., 2019). Cytosolic calcium concentration has an effective role in DC migration and is important for processes such as chemokine receptor expression, cell swelling, and cytoskeletal changes (Crottès et al., 2016). Potassium channels such as the KCa3.1 channels can control the calcium levels in the DCs and the capacity of cell migration (Vandier and Velge-Roussel, 2018). Migration of DCs requires chemokine receptor expression and optimal levels of cytosolic calcium levels, which are regulated by the functioning of these channels (Vandier and Velge-Roussel, 2018). These cytosolic calcium levels are maintained by regulating the membrane potential by the KCa3.1 channels as they hyperpolarize the plasma membrane and favor calcium entry by increasing the driving force for calcium (Vandier and Velge-Roussel, 2018). In DCs, the involvement of KCa3.1 is limited to calcium homeostasis and migration of DCs (Vandier and Velge-Roussel, 2018). Moreover, in T lymphocytes, ion channels have a prominent role in maintaining calcium levels, which are crucial for the proliferation and effector functions of cells (Cahalan and Chandy, 2009). In human T cells, Kv1.3 and KCa3.1 are the principal potassium channels that are important for maintaining the electrochemical driving force (Chirra et al., 2022). The voltage-gated Kv1.3 channel in T cells gets activated by calcium influx mediated by the calcium release-activated channels (CRACs), while the increase in Ca^2+^ activates KCa3.1, which causes efflux of K^+^ ions, thereby maintaining the membrane potential and driving force for the influx of Ca^2+^, as represented in Figure 2 (Teisseyre et al., 2019; Chirra et al., 2022). The expression, function, and role of the K^+^ channel vary as per the activation status and subset of T cells (Beeton and Chandy, 2005; Pérez-García et al., 2018). Upon activation, depending on the T-cell subset, the type of K^+^ channel is upregulated (Beeton and Chandy, 2005; Pérez-García et al., 2018). In resting human T cells, Kv1.3 is expressed more compared to KCa3.1, and a similar effect is observed in activated T_EM_ (Beeton and Chandy, 2005; Pérez-García et al., 2018). In the case of activated central memory T cells (T_CM_), the expression of KCa3.1 is more abundant (Beeton and Chandy, 2005; Pérez-García et al., 2018). Both the K^+^ channels are implicated in proliferation and cytokine production, and the loss of Kv1.3 function is compensated by KCa3.1 channels by maintaining the proliferation and effector function of T cells (Chiang et al., 2017). Apart from this, Kv1.3 channels are involved in the differentiation and regulation of CD8^+^ T cells and CD4^+^ T cells (Chiang et al., 2017).

**FIGURE 3:**
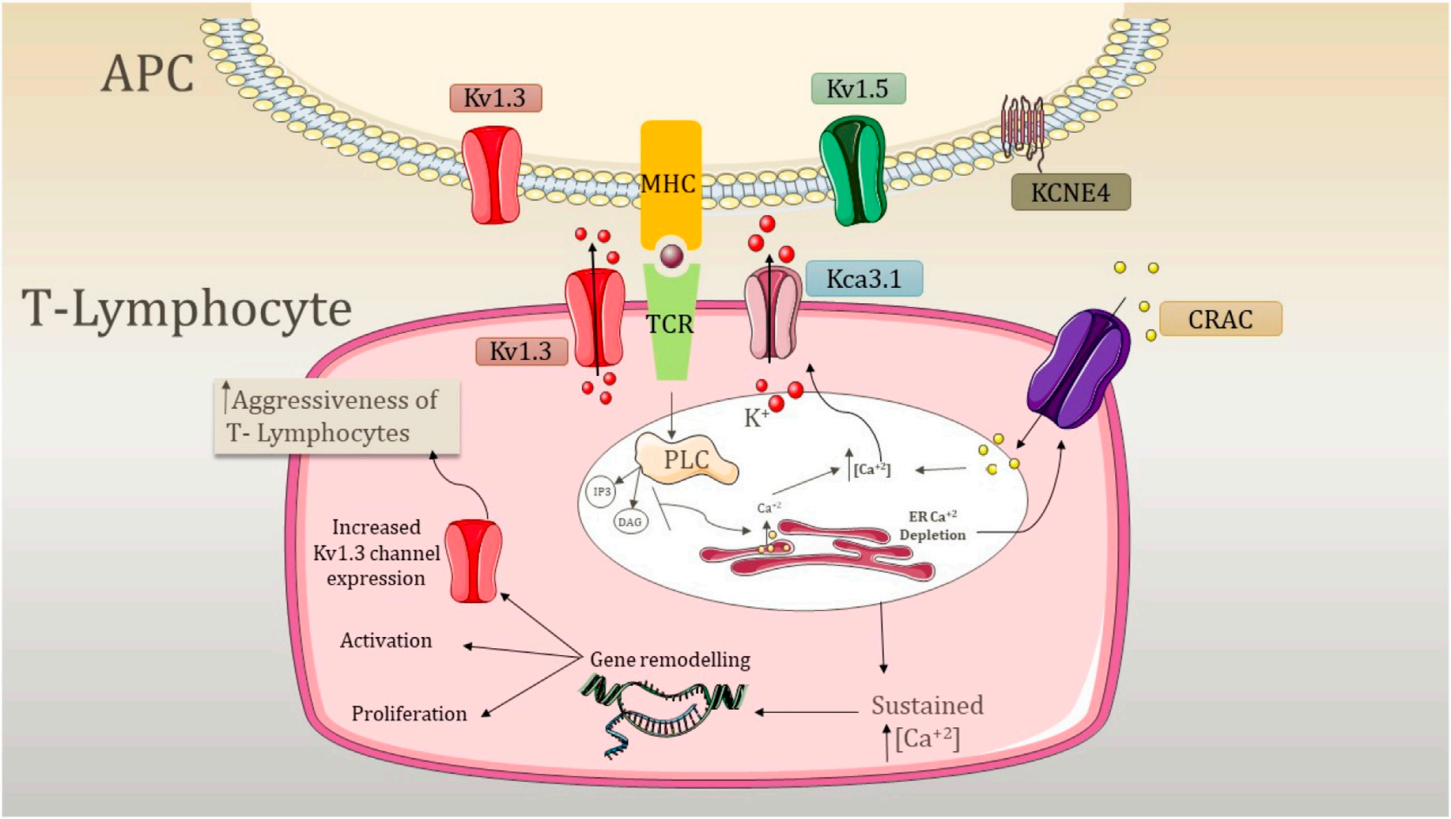
Mechanism underlying the aggression of T lymphocytes due to the activity of potassium channels. APC, antigen presenting cell; PLC, phospholipase C; CRAC, calcium-release-activated Ca^2+^ channel; TCR, T-cell receptor; MHC, major histocompatibility complex; K^+^, potassium ion; IP_3_, inositol triphosphate; DAG, diacylglycerol; Kv1.3, voltage-gated potassium channel 1.3; KCa3.1, calcium-activated potassium channel 3.1; Kv1.5, voltage-gated potassium channel 1.5; and KCNE4, potassium voltage-gated channel subfamily E member 4. The expression of Kv1.3 is shared by both T lymphocytes and APC. APC involves the expression of Kv1.5 and KCNE4, which modulates the expression of Kv1.3. The signal transduction cascade is initiated by the antigen presentation by MHC and TCR recognition, which causes the activation of PLC. PLC further activates IP3 and DAG, resulting in the depletion of internal ER Ca^2+^ stores. This activates CRAC channels and causes depolarization. Kv1.3 opens as a result of depolarization, causing K^+^ efflux through the membrane. In addition, an increase in internal Ca^2+^ concentration activates the KCa3.1 channel, and both K^+^ channels maintain the negative membrane potential required for sustained Ca^2+^ release. This sustained Ca^2+^ release causes the activation of nuclear processes such as activation and proliferation, and an increase of Kv1.3 abundance elevates the response, increasing the aggressiveness of T cells.

FLSs play an essential role in synovial joint destruction as inflammatory mediators (Wu et al., 2021b). Emerging evidence suggests that the mechanism of highly dynamic synoviocytes is linked to the membrane channels in inflamed joints. In RA patients, KCa1.1 is the major channel present in the plasma membrane of FLSs (Ji and Hong, 2019). They play an important role in regulating β1 integrins by maintaining Ca^2+^ homeostasis (Tanner et al., 2017b; Ji and Hong, 2019). Increased integrin ligation is associated with growth factor expression and increased cytokine signaling (Tanner et al., 2017a). KCa1.1 affects the proliferation and activation of T_EM_ cells, which are involved in the progression of RA, while an increased expression of this channel represents the invasiveness of FLSs (Ji and Hong, 2019; Tanner et al., 2019). Research conducted by Friebel et al. indicated that the calcium-activated potassium channel KCa3.1 is functionally active in RA and expressed at the mRNA and protein levels in RA synovial fluids (SFs) (Friebel et al., 2015). They have a regulatory impact on cell proliferation and secretion of pro-inflammatory and pro-destructive mediators (Friebel et al., 2015). Apart from this, they are also involved in the expression and secretion of IL-6, IL-8, MCPI, and the tissue-destructive protease MMP3 (Friebel et al., 2015). K2P5.1 (TWIK-related acid-sensitive potassium channel 2 (TASK2); KCNK5) is a member of the two-pore domain potassium channel family expressed in CD4^+^ T cells (Bittner et al., 2010; 2011). The study performed by Bittner et al. revealed that the expression levels of K2P5.1 in synovial fluid-derived T cells are higher compared to those of peripheral blood T cells (Bittner et al., 2011). K2P5.1 is engaged in T-cell functions such as proliferation and cytokine production (Endo et al., 2015). In addition, there is a correlation between the expression levels of the channel in T lymphocytes with patients suffering from RA, and an increased expression of this channel is observed in RA patients (Bittner et al., 2011). The activation of T lymphocytes is associated with the pathogenesis of RA and the expression of potassium ion channels (Figure 3).

This evidence and hypothesis of the role of potassium channels in the immune system and RA pathogenesis focus on the potassium channels as therapeutic targets in RA. Due to their convincing role in maintaining disease pathogenesis and the progression of RA, potassium channels have a significant opportunity of being used as potential therapeutic targets in RA.

## 5 Potassium channels as therapeutic targets for RA

The conventional therapies for RA include DMARDs and glucocorticoids (GC), which are still a primary line of treatment (Kerschbaumer et al., 2020; Tan and Buch, 2022). This treatment does not provide a complete cure and causes several side effects, and patients’ tolerance to the therapy also varies. Further advancements in recent years in treatment include T- and B-cell-targeted therapies and drugs targeting TNF-α and interleukins, the JAK pathway, and various other targets such as the Toll-like receptors, neuropathways, and dendritic cells, and these treatment options cause specific side effects (Cheung and McInnes, 2017). The above sections explain the role of ion channels in the pathogenesis of RA. Therefore, the ion channels, specifically potassium channels, as therapeutic targets can be a step toward exploring a new therapy for the treatment of RA. Both voltage-gated and calcium-activated potassium channels are involved in the disease progression and pathogenesis of RA, and modulation of these channel activities can be considered a new avenue for the treatment of RA. The evidence for their efficacy is provided in Table 1.

**TABLE 1:** Voltage-gated and calcium-activated potassium blockers for the treatment of RA.

Type	Subtype	Blocker	Outcome	Reference
Voltage-gated	Kv1.3	HsTx1	Inhibits T_EM_ cell activation and attenuates inflammation in autoimmunity	Tanner et al. (2017a)
Potassium channels	Kv1.3	Diclofenac	Inhibits macrophages, T lymphocytes, and IL-2 production	Villalonga et al. (2010)
	Kv1.3	Margatoxin	Attenuates macrophages migration, IL-2 production, and T-cell activity	Villalonga et al. (2010)
	Kv1.3	Charybdotoxin	Attenuates macrophage migration, IL-2 production, and T-cell activity	Villalonga et al. (2010)
	Kv1.3	ShK-186	Decreases the number of affected joints and showed improvement in the radiological and histopathological studies	Beeton et al. (2011)
	Kv1.3	Spiro-azepene	Attenuates T-cell activation	Wulff (2010)
		Oxazolidinediones		
	Kv1.3	Kaliotoxin	Treats delayed-type hypersensitivity in RA patients	Beeton et al. (2001), Wulff (2010)
Calcium-activated	KCa3.1	TRAM-34	It reduced the production of several pro-inflammatory mediators such as IL-6, IL-8, and monocyte chemotactic protein-I (MCP-I)	Friebel et al. (2015)
Potassium channel	KCa1.1	Tetraethyl ammonium (TEA)	Disturbs calcium homeostasis and inhibits the proliferation, invasion, and production of VEGF, IL-8, and pro-MMP 2	Hu et al. (2012)
	KCa3.1	Clotrimazole	Shows beneficial effects such as improved grip strength, pain relief, reduced joint swelling	Wulff et al. (2023)
	KCa1.1	Paxilline	Inhibits the proliferation and production of PIA-FLS and pro-MMP-2	Tanner et al., 2015 (2018)
	KCa1.1	Iberiotoxin	Inhibits the proliferation and production of PIA-FLS and pro-MMP-2	Tanner et al., 2015 (2018)
	KCa3.1	Hydroxychloroquine (HCQ)	Showed a dose-dependent inhibition of THP-1 macrophages, NLRP-3 inflammasomes, IL-1β, and caspase	Eugenia Schroeder et al. (2017)

### 5.1 Voltage-gated potassium channels

Kv1.3 is the most studied and widely explored voltage-gated potassium channel due to its significant role in autoimmunity and the pathogenesis of RA (Serrano-Albarrás et al., 2019). Pieces of evidence suggest that an increased expression of this channel is associated with the pathogenetic roadmap of RA (Serrano-Albarrás et al., 2019). Thus, blockage of this channel with the help of therapeutic agents can be beneficial for the treatment of RA (Serrano-Albarrás et al., 2019).

Studies performed by Tanner et al. showed scorpion venom peptide HsTx1 as a potent inhibitor of the Kv1.3 channel (Tanner et al., 2017b). It is a key regulator of the potassium channel and CCR7^-^ T_EM_ cell activation. Blockage of the Kv1.3 channel using HsTx1 leads to the inhibition of T_EM_ cell activation and attenuates inflammation in autoimmunity (Tanner et al., 2017a). Results from the study showed a reduction in inflammation due to delayed-type hypersensitivity in a pristane-induced (PIA) rat model of RA (Tanner et al., 2017b). The Kv1.3 channel is present in lymphocytes and is an important target for immune modulation (Toldi et al., 2016). An *ex vivo* study performed by Toldi et al. compared the alteration in cytokine production using the selective Kv1.3 blocker margatoxin (MGTX) and found that there was a decrease in the calcium influx of CD4^+^ and Th2 subsets across the study group (Toldi et al., 2016; Xie et al., 2020), while treatment with MGTX, a selective Kv1.3 blocker, did not show any influence on cytokine production (Toldi et al., 2016; Xie et al., 2020). The treatment in an *ex vivo* study with diclofenac, a Kv1.3 channel blocker, showed inhibition of macrophages and T lymphocytes in diseased conditions (Villalonga et al., 2010). It is shown that pharmacological doses of diclofenac attenuate macrophage migration, IL-2 production, and T-cell activity in an LPS-induced model (Villalonga et al., 2010). Similar effects were shown by margatoxin, a Kv1.3 channel blocker, and charybdotoxin, which blocks both the Kv1.3 and KCa3.1 channels (Villalonga et al., 2010). ShK is a polypeptide obtained from the Caribbean sea anemone *Stichodactyla helianthus*, which was used by researchers as a Kv1.3 channel blocker (Beeton et al., 2011). ShK blocks the Kv1.3 channel at picomolar concentrations (Beeton et al., 2006; 2011). Studies have shown that ShK analog ShK-186 at 100 μg/kg body weight showed efficacy in treating RA (Beeton et al., 2011). After inducing the rats with diseased conditions, the results revealed that ShK-186-treated animals had significantly fewer affected joints and showed improvement in radiological and histopathological studies (Beeton et al., 2011). In 2008, a company, Solvay Pharmaceuticals, filed a patent for oxazolidinediones-spiro-azepene as a novel blocker of Kv1.3 potassium channels for the treatment of T-cell-regulated autoimmune diseases such as RA (Wulff, 2010). In addition, kaliotoxin is a molecule with Kv1.3 blocking activity and can be used to treat delayed-type hypersensitivity in RA patients (Beeton et al., 2001; Wulff, 2010). A recently published study showed that dexamethasone blocks the Kv1.3 channel in peripherally circulating CD8^+^ T cells of severely ill patients with COVID-19 (Chimote et al., 2023). This study suggests that steroidal therapies used to treat RA may have an indirect effect on Kv1.3 channels.

### 5.2 Calcium-activated potassium channels

Similar to voltage-gated potassium channels, calcium-activated potassium channels play a crucial role in the immune function and pathogenesis of RA (Lam and Wulff, 2011). The *ex vivo* studies performed by *Friebel et al.* concluded that KCa3.1, a calcium-activated potassium channel, is involved in the pathological maintenance of RA by regulating the cell proliferation and secretion of pro-inflammatory and pro-destructive mediators (Friebel et al., 2015). Since KCa3.1 is significantly expressed and functional in SFs of RA patients, the study focused on the blocking of this channel with TRAM-34, a pore-blocking KCa3.1 inhibitor (Friebel et al., 2015). The results indicated that blocking of KCa3.1 with TRAM-34 was beneficial as it reduced the production of several pro-inflammatory mediators such as IL-6, IL-8, and monocyte chemotactic protein-I (MCP-I) (Friebel et al., 2015). Hu et al. demonstrated that blocking of the KCa1.1 channel with tetraethylammonium (TEA) disturbs the calcium homeostasis and inhibits the proliferation, invasion, and production of VEGF, IL-8, and pro-MMP 2 by RA FLs. Although TEA has the ability to block voltage-gated potassium channels, it was not used clinically due to its non-specificity (Hu et al., 2012). Furthermore, an antifungal agent, clotrimazole, was used in RA due to its ability to block the KCa3.1 channel and showed beneficial effects such as improved grip strength, pain relief, and reduced joint swelling (Wulff et al., 2023). Tanner et al. showed the importance of KCa1.1 channel expression in FLs of patients in PIA- and CFA-induced models of RA (Wulff et al., 2023). In an RA rat model, KCa1.1 channel blockers such as paxilline, TEA, and iberiotoxin were used to block the channel activity, which resulted in an approximately 80% blockage of potassium currents by TEA and paxilline (Tanner et al., 2015; 2018). It has been shown that both paxilline and TEA inhibit the proliferation and production of PIA-FLS and pro-MMP-2, and a similar activity was shown by iberiotoxin (Tanner et al., 2015; 2018). Schroeder et al. used hydroxychloroquine (HCQ) to inhibit KCa3.1 during *in vitro* studies and demonstrated dose-dependent inhibition of IL-1β and caspase 1 by HCQ (Eugenia Schroeder et al., 2017).

These pieces of evidence and results prove the impact of voltage-gated and calcium-activated potassium channels in the pathogenesis and treatment of RA. Furthermore, detailed research is required to improve the specificity of molecules toward the particular ion channel to reduce the side effects.

## 6 Conclusion

RA is an autoimmune inflammatory disorder with serious effects on the wellbeing of patients and the ability to perform daily functions. The interaction between genetic and environmental factors is one of the reasons for the development of RA, while autoimmunity also has a significant contribution to disease progression. The current first-line therapy for RA has several side effects, and some patients may show poor tolerance to the treatment, which increases the limitation of the therapy. Thus, there is a need for new treatment strategies for the treatment of RA with minimal side effects. Several pieces of evidence confirm the involvement of voltage-gated and calcium-activated K^+^ channels in the pathogenesis of RA due to their role in maintaining the immune function and immune cells. This makes K^+^ channels a promising target for the treatment of RA. The studies performed by the researchers demonstrated the effectiveness of potassium channels, specifically Kv1.3, KCa1.1, and KCa3.1, in various models of RA. The blockers of these channels with various pharmacological agents were found to be beneficial in rat models of RA. Although no clinical trial has yet been performed for the effectiveness of Kv1.3 blockers in humans, more research is required regarding these channels. The focus should be on designing more target-specific strategies and molecules to reduce any kind of side effects and create potential therapies. Ultimately, target-specific studies with a greater extent are required for this channel to explore the therapeutic opportunities of potassium channels in the treatment of RA.

## Figures and Tables

**Table:** 

**ACPA**	Antibodies against citrullinated proteins
**cDCs**	Classical DCs
**CRAC**	Calcium release-activated Ca^2+^ channels
**CRP**	C-reactive protein
**DCs**	Dendritic cells
**DMARDs**	Disease-modifying anti-rheumatic drugs
**DTH**	Delayed-type hypersensitivity
**ELN**	Ectopic lymphoid neogenesis
**ESR**	Erythrocyte sedimentation rate
**FcγR**	Fcγ receptor
**FDCs**	Follicular dendritic cells
**FGF**	Fibroblast growth factor
**FLS**	RA-fibroblast-like synoviocytes
**GC**	Glucocorticoids
**IFN-ϒ**	Interferon-ϒ
**ILCs**	Innate lymphoid cells
**K2P5.1**	TWIK-related acid-sensitive potassium channel 2
**MBDA**	Multi-biomarker activity test
**M-CSF**	Macrophage colony-stimulating factor
**MGTX**	Margatoxin
**MHC**	Major histocompatibility complex
**MMPs**	Matrix metalloproteinases
**NK cells**	Natural killer cells
**PB**	Peripheral blood
**pDCs**	Plasmacytoid DCs
**RA**	Rheumatoid arthritis
**RAG**	Recombination activating gene
**RANKL**	Receptor activator of nuclear kappa beta ligand
**RF**	Rheumatoid factor
**ROS**	Reactive oxygen species
**SF**	Synovial fluid
**SNPs**	Single nucleotide polymorphism
**T**_**CM**_	Central memory T cells
**T**_**EM**_	Effector memory T cells
**Tfh**	Follicular helper T cells
**TLR**	Toll-like receptor
**TNF-α**	Tumor necrosis factor-alpha
**Tph**	Peripheral helper T cells
**T**_**regs**_	T-regulatory cells
